# Effectiveness of electroacupuncture for pain after osteosarcoma post surgery: A study protocol of systematic review of randomized controlled trial

**DOI:** 10.1097/MD.0000000000017381

**Published:** 2019-11-01

**Authors:** Chi He, Qing-xi Tang, Ying-xia Li, Kai He, Zhi-ling Hou

**Affiliations:** a Department of Anesthesiology; b Department of Emergency Surgery, First Affiliated Hospital of Jiamusi University, Jiamusi; c Department of Orthopedis, Harbin Second Hospital, Harbin, China.

**Keywords:** effectiveness, electroacupuncture, osteosarcoma, pain, safety

## Abstract

**Background:**

This study will assess the effectiveness of electroacupuncture (EA) for pain in patients with osteosarcoma post surgery (OSPS).

**Methods:**

In this study, we will comprehensively search the following electronic databases from inception to the present without language restrictions: Cochrane Library, EMBASE, MEDLINE, the Cumulative Index to Nursing and Allied Health Literature, the Allied and Complementary Medicine Database, and Chinese Biomedical Literature Database. Two authors will independently carry out study selection, data extraction, and methodological assessments. RevMan 5.3 software will be used for statistical analysis.

**Results:**

The primary outcome is pain intensity. The secondary outcomes consist of event-free survival, overall survival, quality of life, and adverse events.

**Conclusion:**

The findings of this study will provide helpful evidence of EA treatment for patients with OSPS.

**PROSPERO registration number:**

PROSPERO CRD42019146696.

## 1 Introduction

Osteosarcoma is the most common malignant bone tumor among the children and young adult population.^[1–3]^ It has been reported that during the past decades, most patients with such disorder died within 1 year after the diagnosis, and the overall 5-year survival rate was about 10%.^[4,5]^ Many factors are accounted for its prognostic recovery, such as surgical criteria, tumor site, alkaline phosphatase, lactate dehydrogenase, and others.^[6–13]^ Thus, the prognostic rehabilitation often depends on these factors, as well as the different treatments.

Management schedules often include aggressive surgery and multi-agent chemotherapy. As for chemotherapy, a standard regimen of high-dose methotrexate, doxorubicin, and cisplatin is utilized to treat this condition.^[14–17]^ However, it has not improved outcomes in some patients and often brings a wide range of serious adverse events.^[18–20]^ On the other hand, surgery is often used as an effective treatment for osteosarcoma during the past few decades. However, it still had limited efficacy outcomes and high relapse rate, as well as a lot of complications, such as pain after surgery for patients with osteosarcoma. Therefore, more effective therapies with fewer adverse events are urgently to treat these complications, especially for the pain.

Fortunately, electroacupuncture (EA) is reported to treat a variety of pain conditions effectively and safely.^[21–23]^ In addition, 2 previous case reports also reported to utilize EA to treat pain for patients with osteosarcoma, and have achieved very promising effectiveness.^[20,24]^ However, no study systematically assesses the effectiveness and safety of EA for pain in patients with osteosarcoma post surgery (OSPS). Therefore, in this study, we will systematically assess the effectiveness and safety of EA for the treatment of pain in patients with OSPS.

## 2 Methods

### 2.1 Study registration

This study has been registered on PROSPERO (CRD42019146696). It has been reported based on the guidelines of Preferred Reporting Items for Systematic Reviews and Meta-Analysis (PRISMA) Protocol statement.^[25]^

### 2.2 Study selection criteria

#### 2.2.1 Study types

This study will consider randomized controlled trials (RCTs) of EA for pain in patients with OSPS for inclusion. Other studies, including non-clinical trials, non-RCTs will be excluded.

#### 2.2.2 Interventions

Study reporting results of interventions involving EA for pain in patients with OSPS will be included. The controls can be any interventions except EA.

#### 2.2.3 Population

OSPS patients with pain, regardless their race, sex, and age will all be considered for inclusion.

#### 2.2.4 Outcomes

The primary outcome is pain intensity, which can be measured by Visual Analogue Scale or any other pain scales. The secondary outcomes include event-free survival, overall survival, quality of life, as measured by 36-Item Short Form Health Survey or any other relevant scales, and adverse events.

### 2.3 Search strategy

The following electronic databases will be comprehensively searched from their inception to the present without language limitations: Cochrane Library, EMBASE, MEDLINE, the Cumulative Index to Nursing and Allied Health Literature, the Allied and Complementary Medicine Database, and Chinese Biomedical Literature Database. The detailed search strategy for Cochrane Library is shown in Table 1. Similar detailed strategies are also applied to the other electronic databases. Additionally, we will also search dissertations, clinical trials registry, and reference lists of included studies to avoid missing any other potential studies.

**Table 1:**
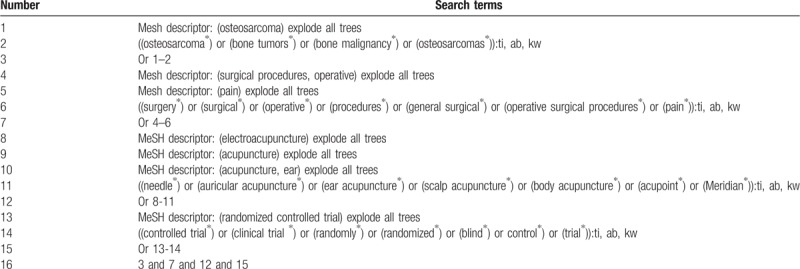
Search strategy for Cochrane Library database.

### 2.4 Study selection

**Figure 1:**
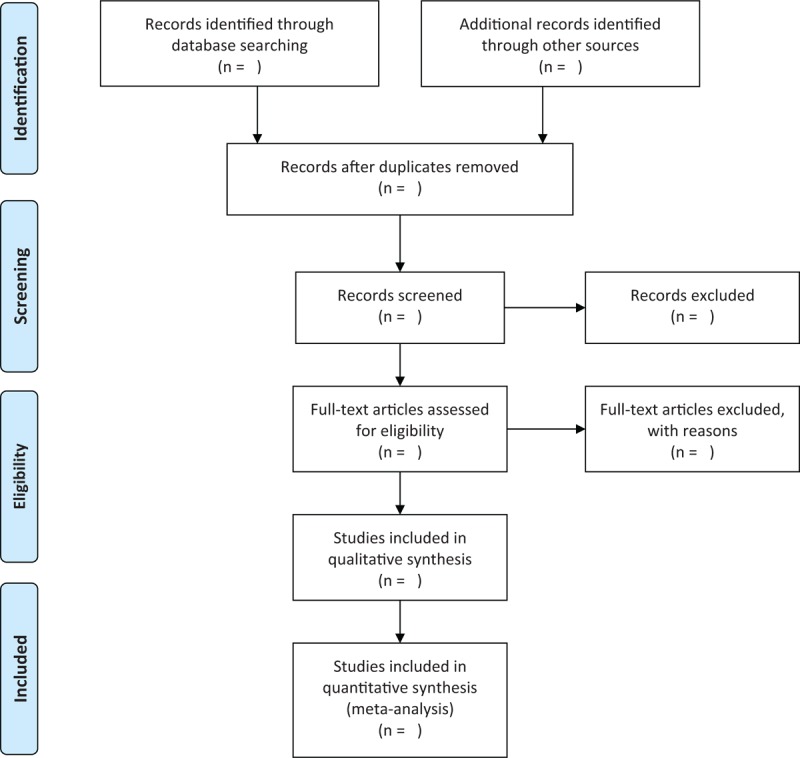
Flowchart of study selection.

Two independent authors will carry out the study selection according to the predefined eligibility criteria. Any disagreements will be solved by a third author through discussion. All literature records will be scanned by reading titles and abstracts initially. Then, all irrelevant literature records will be excluded. After that, the remaining records will be read by full-texts. The study selection process will be presented in a PRISMA flow chart in Figure 1.

### 2.5 Data extraction and management

Two authors will independently extract all information according to the pre-designed data extraction sheet. Any divergences regarding the data extraction between 2 authors will be resolved by a third author through discussion. The extracted information comprises of title, authors, published year, patients’ information, sample size, study design, study methods, treatment details, outcome measurements, and adverse events.

### 2.6 Risk of bias assessment

Risk of bias of all included studies will be assessed by using Cochrane Risk of Bias Tool. It consists of 7 aspects, and each aspect is further classified as high, unclear, and low risk of bias. Two authors will independently assess the risk of bias for all included studies. Any divisions will be tackled by a third author through discussion in this study.

### 2.7 Treatment effect measurement

In this study, mean difference or standardized mean difference with 95% confidence intervals will be used for expression of continuous data, and risk ratio with 95% confidence intervals will be utilized for expression of dichotomous data.

### 2.8 Missing data dealing with

If there is missing, insufficient or unclear data, we will contact primary authors to require those data. If we can not get those data, the available data will be analyzed and potential impacts of those data will be discussed.

### 2.9 Heterogeneity assessment

In this study, *I*^*2*^ test will be used to identify the heterogeneity among studies. *I*^2^ ≤ 50% indicates reasonable heterogeneity, while *I*^2^ > 50% demonstrates substantial heterogeneity.

### 2.10 Data synthesis

We will use RevMan 5.3 software to pool and analyze all outcome data. We will conduct meta-analysis if there are 2 or more eligible studies on the same outcome measurements with reasonable heterogeneity (*I*^2^ ≤ 50%). If heterogeneity is reasonable, a fixed-effects model will be applied to pool the data, and meta-analysis will be carried out with sufficient studies. If heterogeneity is substantial (*I*^2^ > 50%), a random-effects model will be used to pool the data, and subgroup analysis will be conducted. If there is still significant heterogeneity, and data will not be pooled, meta-analysis will not be carried out. Instead, we will only report narrative summary for the outcome results.

### 2.11 Subgroup analysis

We will carry out subgroup analysis based on the differences of patient characteristics, treatments, controls, and outcome measurements.

### 2.12 Sensitivity analysis

We will also carry out sensitivity analysis to check the robustness of outcome results by removing low methodological quality studies.

### 2.13 Publication bias

In this study, funnel plot and Egger regression will be carried out to check if there is any reporting bias.^[26,27]^

## 3 Discussion

Though a variety of clinical studies have reported its effectiveness for OSPS treatment, no study has been conducted systematically to assess the efficacy and safety of EA for treating OSPS. This study will apply rigorous methodology to explore studies reporting the outcome results of EA for the treatment of patients with OSPS. The results of this study will inform our understanding of the effect of EA in treating OSPS outcomes. In addition, this study may also provide useful evidence for patients and clinicians.

## Author contributions

**Conceptualization:** Chi He, Qing-xi Tang, Ying-xia Li, Kai He.

**Data curation:** Chi He, Kai He.

**Formal analysis:** Chi He, Qing-xi Tang, Ying-xia Li, Kai He.

**Methodology:** Chi He, Qing-xi Tang, Ying-xia Li.

**Resources:** Chi He, Ying-xia Li, Kai He.

**Software:** Chi He, Qing-xi Tang, Ying-xia Li, Kai He.

**Validation:** Chi He, Kai He.

**Visualization:** Chi He, Qing-xi Tang, Ying-xia Li.

**Writing – original draft:** Chi He, Qing-xi Tang, Ying-xia Li, Kai He.

**Writing – review & editing:** Chi He, Qing-xi Tang, Ying-xia Li, Kai He.
